# Periostin is frequently overexpressed and enhances invasion and angiogenesis in oral cancer

**DOI:** 10.1038/sj.bjc.6603431

**Published:** 2006-10-24

**Authors:** B S M S Siriwardena, Y Kudo, I Ogawa, M Kitagawa, S Kitajima, H Hatano, W M Tilakaratne, M Miyauchi, T Takata

**Affiliations:** 1 Department of Oral Maxillofacial Pathobiology, Division of Frontier Medical Science, Graduate School of Biomedical Sciences, Hiroshima University, 1-2-3 Kasumi, Minami-Ku, Hiroshima 734-8553, Japan; 2 Department of Oral Pathology, Faculty of Dental Sciences, University of Peradeniya, Sri Lanka; 3 Center of Oral Clinical Examination, Hiroshima University Hospital, Hiroshima University, Hiroshima 734-8553, Japan

**Keywords:** periostin, oral cancer, invasion, metastasis, angiogenesis

## Abstract

Oral squamous-cell carcinoma (OSCC) is one of the most common types of human cancer. Typically OSCC cells show persistent invasion that frequently leads to local recurrence and distant lymphatic metastasis. We previously identified Periostin as the gene demonstrating the highest fold change expression in the invasive clone by comparing the transcriptional profile of parent OSCC cell line and a highly invasive clone. Here, we demonstrated that Periostin overexpression enhanced invasiveness in oral cancer cell lines. To know the role of Periostin in invasion, angiogenesis and metastasis in OSCC cases, we first examined the expression of Periostin mRNA in 31 OSCC cases by RT–PCR and Periostin protein in 74 OSCC cases by immunohistochemistry. Then, we compared the Periostin expression with invasion pattern, metastasis and blood vessel density. Periostin mRNA and protein overexpression were frequently found in OSCC cases and Periostin expression was well correlated with the invasion pattern and metastasis. Moreover, blood vessel density of Periostin-positive cases was higher than those of Periostin-negative cases. Interestingly, recombinant Periostin enhanced capillary formation *in vitro* in a concentration-dependant manner. In summary, these findings suggest that Periostin may promote invasion and angiogenesis in OSCC, and that Periostin can be a strong marker for prediction of metastasis in oral cancer patients.

Cancers of the oral cavity accounts for 274 000 cases in 2002, with almost two-thirds of them in men (Parkin *et al*, 2005). Squamous-cell carcinoma of the head and neck is a heterogeneous disease with distinct patterns of presentation and behaviour (Forastiere *et al*, 2001). Individual tumour behaviour varies greatly, with some tumours growing slowly and others rapidly or metastasising early. Despite aggressive and multidisciplinary treatment approaches, including preoperative or postoperative chemotherapy and/or radiotherapy with reconstructive surgery, there has been no significant improvement in 5-year survival over the past 20 years (Friedlander, 2003). Review of literature indicated that the most important factor for high mortality rate is the advanced stage of the disease at the time of diagnosis and treatment. The molecular genetic background facilitating this behaviour is an area of great interest, especially in regard to invasion. Like most epithelial cancers, oral squamous-cell carcinoma (OSCC) develops through the accumulation of multiple genetic and epigenetic alterations in a multistep process. The most important prognostic indicator of the patients with OSCC is metastasis to cervical lymph nodes or distant organs (Regezi and Sciubba, 1989). The process of metastasis consists of sequential and selective steps, including proliferation, induction of angiogenesis, detachment, motility, invasion into circulation, aggregation and survival in the circulation, cell arrest in distant capillary beds and extravasation into organ parenchyma (Fidler, 1991). Recent molecular studies have advanced our understanding of the disease and provided a rationale to develop novel strategies for early detection, classification, prevention and treatment. Attempts to identify the genes involved in the metastasis are pivotal for the early prediction of OSCC behaviour. However, the identity and time of onset of the alterations that endow cancer cells with these metastatic functions are largely unknown.

We previously established OSCC cell line from a metastatic lymph node (Kudo *et al*, 2003). Moreover, we isolated highly invasive clone from this cell line by using *in vitro* invasion assay method (Kudo *et al*, 2004). Then, we compared the transcriptional profile of parent OSCC cells and a highly invasive clone by microarray analysis in order to identify the genes that differ in their expression. We identified Periostin (osteoblast-specific factor 2 (fasciclin I-like)) as the gene demonstrating the highest fold change expression in the invasive clone. Periostin is a secreted protein, which has been suggested to function as a cell adhesion molecule for preosteoblast and to participate in osteoblast recruitment, attachment and spreading (Takeshita *et al*, 1993; Horiuchi *et al*, 1999). As we expected, Periostin overexpression enhanced invasion and anchorage-independent growth in OSCC cells (Kudo *et al*, 2006). Interestingly, Periostin-overexpressing cells spontaneously metastasised to cervical lymph nodes and to the lung through their aggressive invasiveness in an orthotopic mouse model of OSCC (Kudo *et al*, 2006). Bao *et al* (2004) also demonstrated that a colon cancer cell line with low metastatic potential engineered to overexpress Periostin displayed a striking phenotype of greatly accelerated tumour metastatic growth as xenografts in the animal model system of metastasis. Previous studies have shown that Periostin promotes metastasis and enhances angiogenesis in breast and colon cancers (Bao *et al*, 2004, Shao *et al*, 2004). These findings indicate that (i) Periostin overexpression may be frequently observed in various types of cancer, and (ii) Periostin may play an important role in invasion, angiogenesis and metastasis, as demonstrated by using cell lines and mice model. However, it is still unclear whether Periostin promotes invasion, angiogenesis and metastasis in actual cancer cases. In the present study, therefore, we examined the expression of Periostin and correlated it with invasion pattern, metastasis and angiogenesis in OSCC cases.

## MATERIALS AND METHODS

### Cell culture

OSCC cell lines, HSC4 and Ca9-22 were provided by Japanese Collection of Research Bioresources Cell Bank. They were maintained in RPMI-1640 (Nissui Pharmaceutical Co., Tokyo, Japan) supplemented with 10% heat-inactivated FBS (Invitrogen, San Diego, CA, USA) and 100 U ml^−1^ penicillin–streptomycin (Invitrogen) under conditions of 5% CO_2_ in air at 37°C. MSCC-1 cell line was previously established in our laboratory (Kudo *et al*, 2003). This cell line was maintained in keratinocyte-SFM (Invitrogen) under a condition of 5% CO_2_ in air at 37°C.

### Retroviral-mediated gene transfer

Packaging GP-293 cells (Clontech, Palo Alto, CA, USA) were transfected with retroviral plasmid encoding a hexa-histidine-tagged Periostin cDNA according to the manufacturer's instructions. A hexa-histidine-tagged Periostin cDNA was kindly provided by Dr X-F Wang (Duke University, Durham, NC, USA). After 48 h of transfection, the virus-containing medium was collected and supplemented with 8 *μ*g ml^−1^ polybrene (Sigma, St Louis, MO, USA). Then, the culture medium of the target cells was replaced with this viral supernatant for 24 h. This infection process was repeated for a second time after a 12 h recovery in normal medium. The stable clones were obtained by puromycin selection (1 *μ*g ml^−1^) in the culture medium.

### *In vitro* invasion assay

*In vitro* invasion assay was performed as described previously (Kudo *et al*, 2006). Briefly, invasion was measured by use of a 24-well cell culture insert with 8 *μ*m pores (3097, Falcon, Becton Dickinson, Franklin Lakes, NJ, USA). The filter was coated with 20 *μ*g of EHS extract (Iwaki Garasu, Tokyo, Japan), which was the reconstituted basement membrane substance. The lower compartment contained 0.5 ml of serum-free medium. After trypsinisation, 1.5 × 10^5^ cells were resuspended in 100 *μ*l of serum-free medium and placed in the upper compartment of the cell culture insert for 18 h. After incubation, we collected the penetrating cells onto the lower side of the filter to isolate highly invasive clones by the method of Kalebic *et al* (1998) with minor modifications. To examine the invasiveness, cells were fixed with formalin and stained with haematoxylin. The invasiveness of the cells was determined by counting of the penetrating cells onto the lower side of the filter through the pores under a microscope at × 100 magnification. We assayed three times and randomly selected three fields were counted for each assay.

### Patients and tissue specimens

OSCC specimens were obtained from 31 patients who underwent surgery at Dental Hospital (Peradeniya, Sri Lanka). These tissue specimens were immediately frozen and stored in −80°C. Informed consent was obtained from all patients for this study. Seventy-four paraffin-embedded tumour tissues were collected from the archives of the same hospital for immunohistochemical staining. Clinical details and lymph node metastasis was gathered from surgical records of the patients.

### Reverse–transcription polymerase chain reaction (RT–PCR)

Total RNA was isolated from tumour tissues using the RNeasy Mini Kit (Qiagen, Hilden, Germany). Preparations were quantified and their purity was determined by standard spectrophotometric methods. cDNA was synthesised from 1 *μ*g total RNA according to the ReverTra Dash (Toyobo Biochemicals, Tokyo, Japan). Two pairs of primer sequences were as follows: human Periostin, 5′-GATGGAGTGCCTGTGGAAAT-3′ (forward) and 5′-AACTTCCTCACGGGTGTGTC-3′ (reverse) (product size, 239 bp); human GAPDH, 5′-TCCACCACCCTGTTGCTGTA-3′ (forward) and 5′-ACCACAGTCCATGCCATCAC-3′ (reverse) (product size, 450 bp); human keratin 5, 5′-ACAGAAGCCGAGTCCTGGTA-3′ (forward) and 5′-AGATTGGCGCACTGTTTCTT-3′ (reverse). Aliquots of total cDNA were amplified with 1.25 U of r*Taq*-DNA polymerase (Qiagen), and amplifications were performed in a PC701 thermal cycler (Astec, Fukuoka, Japan) for 30 cycles after an initial 30 s denaturation at 94°C, annealed for 30 s at 60°C and extended for 1 min at 72°C in all primers. The amplification reaction products were resolved on 1.5% agarose/TAE gels (Nacalai tesque Inc., Kyoto, Japan), electrophoresed at 100 mV and visualised by ethidium-bromide staining.

### Histopathological and immunohistochemical analyses

The tumour tissues were fixed in 10% formalin, embedded in paraffin and cut into 4 *μ*m thick sections. The sections were stained with haematoxylin and eosin for histological examination. All 74 tumours were graded according to the classification described by Jacobsson *et al* (1973) as patterns I, II, III and IV. For immunohistochemical examination of Periostin, a modification of the streptavidin–biotin–peroxidase-complex (SABC) method was used. The tissue sections were deparaffinised and rehydrated in a graded series of alcohols. Endogenous peroxidase activity was blocked with 0.3% H_2_O_2_ for 30 min. The sections were microwaved three times for 5 min each in citrate phosphate buffer (pH 6.0) for antigen retrieval. The sections were then incubated with 10% normal bovine serum albumin in phosphate-buffered saline (PBS) for 10 min to block nonspecific background staining. A polyclonal anti-Periostin antibody was generated by immunising the rabbits with specific peptides (EGEPEFRLIKEGETC) for Periostin and purified through an affinity column. Polyclonal antibody against Periostin was applied as a primary antibody at a dilution of 1 : 100 and incubated at 4°C overnight. After washing with PBS, biotinylated goat anti-rabbit IgG was applied to the section, which were then incubated for 1 h at room temperature. Primary antibody was visualised with diaminobenzidine. Sections were counterstained with haematoxylin, dehydrated and mounted. Periostin expression was graded as positive (over 10% of tumour cells showed strong or diffuse immunopositivity) and negative (less than 10% of the tumour cells showed weak or focal immunopositivity or no staining) by consideration of percentage of positive cells and the overall intensity of immunoreactivity. A cutoff of 10% Periostin-positive cells was applied to separate positive and negative expressors. Maximally selected Fisher's exact test was used to demonstrate that 10% was a good cutoff point (data not shown). Three pathologists (SS, YK and IO) made all the assessments.

### Assay for blood vessel density

CD34 is an antigen present in haematopoietic progenitor cells and endothelial cells. Anti-CD34 antibody is a highly sensitive marker for endothelial cell differentiation and has also been studied as a marker for vascular tumours. To investigate the relation between angiogenesis and Periostin, we have stained all OSCC cases with CD34 endothelial marker (Novocastra Laboratories Ltd, Newcastle, UK) by SABC method. In order to assess blood vessel density, we performed histomorphometric analysis. Three representative photomicrographs (areas where Periostin is positive including invasive front) were taken from each case stained for CD34. First, we went through all the sections stained for Periostin and CD34 antibodies. The area that was selected had the following criteria: (i) expression of Periostin, (ii) included invasive front and (iii) high number of blood vessels. In some tumours, it was difficult to take all above three criteria in one field, as Periostin-positive cells were little far from the invasive front. Photographs of those tumours were taken close proximal to Periostin-expressing area, including invasive front. For Periostin-negative cases, three areas from invasive front were selected. Any brown staining endothelial cell or endothelial cell cluster, with or without a lumen, was considered as a single, countable blood vessel. Stromal area was quantitatively analysed using the digital image (Adobe Photoshop, Adobe and Scion Image software, Scion). From each figure, total counts of blood vessels per stromal area were taken and the average was calculated. The results were then compared with Periostin expression.

### Generation of recombinant Periostin

Full-length human Periostin cDNA was subcloned into pIZ/V5-His vector (Invitrogen). pIZ/V5-His vector containing Periostin was transfected into High-Five insect cells by using Cellfectin reagent (Invitrogen). Stable clones were obtained by Zeocin selection in the culture medium. A Ni-nitrilotriacetic acid column was used to purify recombinant Periostin according to the manufacturer's instructions (Invitrogen).

### *In vitro* angiogenesis by human umbilical vein endothelial cells

Because the Periostin-positive tumours showed higher number of vessels, we examined *in vitro* angiogenesis by using recombinant Periostin. An angiogenesis assay kit obtained from Kurabo (Osaka, Japan) was used according to the manufacturer's instructions with minor modification (Bishop *et al*, 1999). Human umbilical vein endothelial cells were treated with different concentrations of recombinant Periostin protein (0, 50, 100 and 250 ng ml^−1^) and changed media every 3 days. We examined 3 wells data point in a single experiment. After 12 days, the cells were fixed at room temperature with cold 70% ethanol for 30 min. The cells were incubated with the anti-human CD31 antibody for 1 h at 37°C, and further with an alkaline phosphatase-conjugated goat anti-mouse IgG antibody. Visualisation was achieved with 5-bromo-4-chloro-3-indolyl phosphate-nitroblue tetrazolium. Tubule score was estimated with the Chalkley count method under a bright-field microscope (Fox *et al*, 1995).

### Statistical analysis

Possible correlation between variables of the analysed tumour samples was tested for association by the Fisher's exact test. For the correlation between Periostin expression and blood vessel density, statistical significance was measured by the Welch test. For *in vitro* angiogenesis assay, statistical significance of tubule score was also measured by the Welch test. A *P*-value <0.05 was required for significance.

## RESULTS

### High expression of periostin is well correlated with invasion pattern and metastasis in OSCC

**Figure 1:**
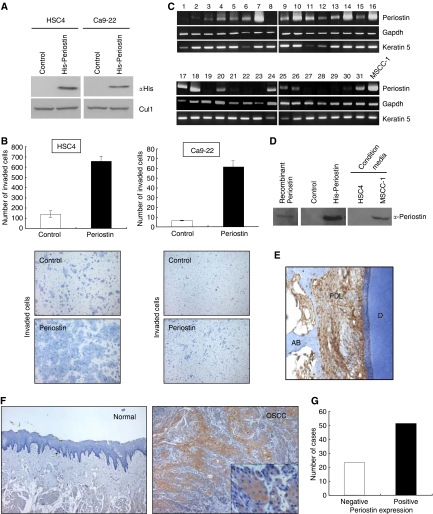
Expression of Periostin in OSCC. (**A**) Overexpression of Periostin in OSCC cells. Using retroviral plasmid encoding a hexa-histidine-tagged Periostin cDNA, infection was performed in HSC4 and Ca9–22 cells, which did not express Periostin. Ectopic expression of Periostin was examined by immunobloting with anti-His antibody. The whole lysates from all samples were blotted with Cul1 for loading control. (**B**) Invasion of Periostin-overexpressing OSCC cells. The invasiveness of the cells was determined by *in vitro* invasion assay as described in ‘Materials and Methods’. Graph shows number of invaded cells after 18 h in control and Periostin-overexpressing OSCC cells, HSC4 and Ca9–22. Figures show the stained (haematoxylin) lower side of the filter where the cells penetrated. Higher number of cells was observed in Periostin-expressed cells. (**C**) Expression of Periostin mRNA in OSCC. Periostin expression was examined by RT–PCR. Some tumours expressed very higher levels of Periostin. Keratin 5 was used as a loading control for the per cent content of epithelial cells. (**D**) Rabbit polyclonal Periostin antibody recognises recombinant, exogenous and secreted Periostin. Expression of Periostin was examined by Western blot analysis in recombinant Periostin itself, His-Periostin-overexpressing HSC4 cells and condition media of HSC4 and MSCC-1 cells. (**E**) Immunohistochemical expression of Periostin in periodontal ligament (PDL) as a positive control (× 200). D, dentin; AB, alveolar bone. (**F**) Immunohistochemical expression of Periostin in OSCC. Representative cases of Periostin expression in normal epithelium (normal) and OSCC are shown. Normal epithelium is completely negative for Periostin compared to OSCC, where most of the tumour cells expressed Periostin (× 40). (**G**) The graph shows immunohistochemical expression of Periostin in 74 OSCC cases. The number of OSCC was plotted against the expression of Periostin. Sixty-nine per cent of them are positive for Periostin.

**Figure 2:**
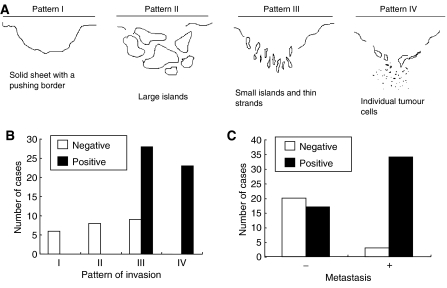
Correlation between Periostin expression and invasion pattern in OSCC. (**A**) Diagrammatic illustration of pattern of invasion, which was first described by Jacobsson *et al* (1973). Pattern I shows a solid sheet of tumour with a pushing border and the pattern II shows large tumour islands. Pattern III shows thin strands of tumour and small tumour islands and pattern IV shows individual tumour cells. (**B**) Bar chart showing the relationship between expression of Periostin and pattern of invasion. Patterns I and II are completely negative for Periostin in contrast with pattern IV where all tumours showed positive results. (**C**) Relationship between Periostin expression and metastasis. Most of lymph node metastatic tumours expressed Periostin.

We previously identified Periostin as an invasion-promoting factor of OSCC by comparing the transcriptional profile between parent OSCC cells and a highly invasive clone (Kudo *et al*, 2006). To confirm whether Periostin can enhance the invasive activity of oral cancer cells, we examined the invasiveness by *in vitro* invasion assay in Periostin transfectant OSCC cell lines (Figure 1A and B). Periostin overexpression strongly enhanced the invasive activity in HSC4 and Ca9-22 cells (Figure 1B). Next, in order to identify the role of periostin in invasion and metastasis in OSCC, we examined Periostin mRNA by RT–PCR. As shown in Figure 1C, 68% (21 of 31) of OSCC cases expressed higher levels of Periostin mRNA. Moreover, we examined 74 OSCC tissue sections by immunohistochemical staining with an antibody against human Periostin. We checked the specificity of polyclonal Periostin antibody. Polyclonal Periostin antibody recognised recombinant Periostin protein, His-Periostin-transfected cells and secreted Periostin in a conditioned media of Periostin-overexpressing cells by Western blot analysis (Figure 1D). Then, by using this antibody, we confirmed the immunohistochemical expression of Periostin in human periodontal ligament in similar to previous reports (Figure 1E). Periostin expression was not observed in normal oral mucosae, whereas OSCC cells expressed Periostin at higher levels (Figure 1F). Similar to RT–PCR analysis, high expression of Periostin was frequently observed in OSCC cases (69%, 51 out of 74 cases) (Figure 1F). Then, we compared Periostin expression with invasion pattern and metastasis in 74 OSCC cases. Jacobsson's classification (patterns I–IV) was used for evaluation of invasion patterns, as shown in Figure 2A(Jacobsson *et al*, 1973). In 74 OSCC cases, there were 6, 8, 37 and 23 cases of patterns I, II, III and IV, respectively. As described in the literature, pattern IV has higher metastatic rate (Unal *et al*, 1999; Sawair *et al*, 2003). Interestingly, patterns I and II were completely negative for Periostin, in contrast with pattern IV, where all cases expressed Periostin (Figure 2B). The correlation between Periostin expression and invasion pattern was statistically significant (*P*<0.005) (Table 1). Interestingly, Periostin expression was significantly correlated with metastasis (*P*<0.005) (Table 1). Most of primary tumours, which showed metastasis were positive for Periostin (Figure 2C).

### Correlation between Periostin expression and angiogenesis

**Figure 3:**
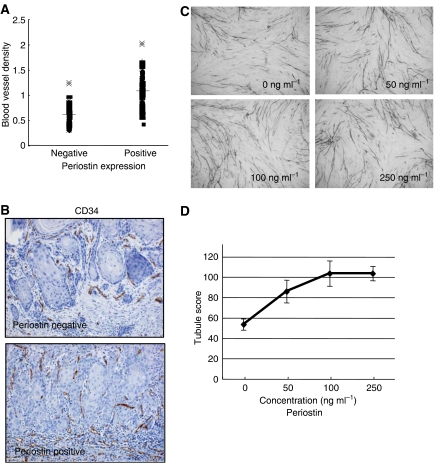
Correlation between Periostin expression and blood vessel density. (**A**) Blood vessel density is plotted against Periostin expression. First, we have stained all OSCC cases with CD34 endothelial marker. For evaluating blood vessel density, we performed histomorphometric analysis as described in Materials and Methods. From three representative areas, total count of blood vessels per stromal area was quantitatively analysed and the average was calculated. For the correlation between Periostin expression and blood vessel density, statistical significance was measured by the Welch test. Periostin expressed tumours showed higher vascular density compared to negative tumours and the results were significant statistically. (**B**) Immunohistochemical expression of CD34. Representative cases of CD34 expression are shown. Periostin-positive tumour shows higher number of vessels (× 100). (**C**) Periostin enhanced capillary formation *in vitro*. Representative areas of capillary formation by Periostin treatment (0, 50, 100 and 250 ng ml^−1^) are shown (× 40). An angiogenesis assay kit was used according to the manufacturer's instructions with minor modification (Fox *et al*, 1995). Human umbilical vein endothelial cells were treated with different concentrations of recombinant Periostin protein (0, 50, 100 and 250 ng ml^−1^) and changed media every 3 days. After 12 days, the cells were fixed and stained with anti-human CD31 antibody as described in Materials and Methods. Tubule score was analysed under a bright-field microscope. (**D**) The graph shows average tubule score after Periostin treatment. The values represent means of tubule score±s.d. based on 3 wells data point in a single experiment. Tubule score was evaluated by the Chalkley count method (Sawair *et al*, 2003).

Thus, we found a possible correlation between Periostin expression and invasion pattern and metastasis. The expression of Periostin in various types of cancers suggests that Periostin may be intimately associated with the progression of tumour development. In addition, we previously demonstrated that Periostin-overexpressing OSCC cells spontaneously metastasised to cervical lymph nodes and lung by orthotopic implantation into the tongue (Kudo *et al*, 2006). As angiogenesis is an important process of metastasis, we examined the correlation between Periostin expression and blood vessel density. We performed an immunohistochemical analysis on tumour sections by utilising an antibody against vascular endothelial cell marker CD34, an assay commonly used to detect the presence of vascular endothelial cells. We performed histomorphometric analysis in order to assess the blood vessel density. Then, we compared blood vessel density with Periostin expression (Figure 3A). Interestingly, the tumours with Periostin expression significantly showed higher number of vessels (high blood vessel density) than those without Periostin expression (*P*<0.005) (Figure 3B). The average of blood vessel density was 0.66±0.2 and 1.08±0.3 in Periostin-negative and -positive cases, respectively (Table 1).

To address the above immunohistochemical results, we examined whether Periostin promotes capillary formation by *in vitro* angiogenesis assay. The number and the length of tubules were higher with high concentration of recombinant Periostin protein (Figure 3C). Tubule score was 53.3±5.6, 85.7±11.1, 103.3±12.4 and 103.3±7.1 after 0, 50, 100 and 250 ng ml^−1^ of Periostin treatment, respectively (Figure 3D). Overall, Periostin promotes capillary formation in a concentration dependant manner.

## DISCUSSION

Periostin contains an N-terminal secretory signal peptide, followed by a cysteine-rich domain, four internal homologous repeats and a C-terminal hydrophilic domain. Periostin is originally identified from osteoblasts and functions as a cell adhesion molecule for preosteoblast and to participate in osteoblast recruitment, attachment and spreading (Takeshita *et al*, 1993; Horiuchi *et al*, 1999). Previous studies showed that the expression of Periostin is upregulated in various types of cancer, including head and neck (Gonzalez *et al*, 2003), colon (Bao *et al*, 2004, Tai *et al*, 2005), breast (Shao *et al*, 2004), lung (Sasaki *et al*, 2001c) and ovarian cancer (Gillan *et al*, 2002). Here, we also found that 68% of OSCC cases expressed Periostin mRNA and 69% of OSCC cases expressed Periostin protein. Cumulative findings suggest that high expression of Periostin may be a common event of tumour development in cancer. However, most of previous studies did not clarify the relationship between Periostin expression and clinicopathological findings.

We identified Periostin as an invasion promoting factor by microarray analysis and demonstrated that Periostin promoted invasion and metastasis in OSCC by *in vitro* and *in vivo* studies using cell lines and mice (Kudo *et al*, 2006). Here, we confirmed that exogenous Periostin expression strongly enhanced invasive activity in oral cancer cells (Figure 1A and B). On the other hand, Bao *et al* (2004) identified Periostin as a metastasis-related gene by differential display analyses using mRNA samples isolated from normal colon tissue, primary colon cancer and metastatic tumour in the liver derived from the same patient. They found that Periostin promoted metastasis in colon cancer by both preventing stress-induced apoptosis in the cancer cells and augmenting endothelial cell survival to promote angiogenesis (Bao *et al*, 2004). Same group also found that Periostin enhanced VEGF receptor Flk-1/KDR expression in endothelial cells through integrin *α*v*β*3-FAK-mediated signalling pathway (Shao *et al*, 2004). Taken together, we thought that Periostin might be involved in invasion, angiogenesis and metastasis in OSCC. However, it is still unclear whether Periostin is involved in invasion and metastasis in actual cancer cases. In the present study, we demonstrated the possible correlation between Periostin expression and clinicopathological findings including invasion pattern and metastasis in OSCC cases. In fact, 14 of 23 (61%) cases showed patterns I and II (a solid sheet of tumour with a pushing border and large tumour islands) and no cases showed pattern IV in Periostin-negative cases, suggesting that Periostin-negative cells may not be able to detach from tumour nests. We also found Periostin expression was well correlated with blood vessels density. Interestingly, Periostin-negative cases showed lower blood vessel density in comparison with Periostin-positive cases. In addition, recombinant Periostin enhanced capillary formation by *in vitro* angiogenesis assay. These findings strongly suggest that Periostin may promote angiogenesis as well as invasion of OSCC cells.

The four internal repeats region of Periostin share a homology with the axon guidance protein FAS1, containing sequences that allows binding of integrins and glycosaminoglycans *in vivo* (Elkins *et al*, 1990). FAS1 domains of *β*ig-h3, which shares a significant structural homology with Periostin, bear motifs interacting with integrins *α*3*β*1 and *α*v*β*5 (Kim *et al*, 2000, 2002) and mediate endothelial cell adhesion and migration via integrin *α*v*β*3 (Nam *et al*, 2003). Similar to *β*ig-h3, we also previously found that interference with the function of integrins by specific anti-*α*v*β*3 and anti-*α*v*β*5 integrin antibodies had an effect on the ability of Periostin to mediate cell adhesion in OSCC cells (Kudo *et al*, 2006). These findings strongly suggest that FAS1 domain of Periostin may be important for binding to integrins both in cancer cells and endothelial cells.

**Figure 4:**
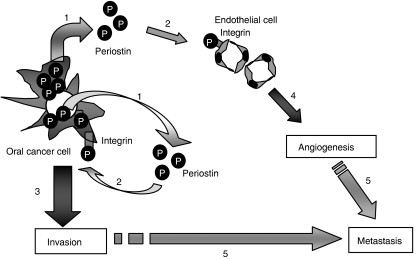
Hypothetic illustration of the role of Periostin in OSCC. We hypothesise that invasion and angiogenesis promoted by Periostin may lead to metastasis of OSCC through the following steps: (1) OSCC cells with high expression of Periostin secrete Periostin. (2) Secreted Periostin binds to integrins both in OSCC cells and endothelial cells. This binding inhibits cell–ECM interaction in OSCC cells. (3) Interaction between Periostin and integrins promotes invasion through inhibition of interaction between integrins and ECM and/or activation of intracellular signal in OSCC cells. (4) Interaction between Periostin and integrins promotes angiogenesis in endothelial cells. (5) Invasion and angiogenesis leads to metastasis.

Taken together with previous and present studies, we hypothesise that invasion and angiogenesis promoted by Periostin may lead to metastasis of OSCC through the following steps: (i) Periostin-overexpressing OSCC cells secrete Periostin, (ii) secreted Periostin binds to integrins both in OSCC cells and endothelial cells, (iii) interaction between Periostin and integrins promotes invasion through inhibition of interaction between integrins and ECM and/or activation of intracellular signal in OSCC cells, (iv) interaction between Periostin and integrins promotes angiogenesis in endothelial cells and (v) invasion and angiogenesis leads to metastasis (Figure 4). Overall, our present findings suggest that Periostin can be a useful marker to predict metastasis in OSCC. It has been reported that serum levels of Periostin were elevated in patients with breast cancer, nonsmall-cell lung cancer and thymoma (Sasaki *et al*, 2001a, 2001b and 2003). In fact, we detected Periostin in condition media of oral cancer cell lines with high expression of Periostin (Kudo *et al*, 2006). Therefore, we can imagine that secreted Periostin from OSCC cells can probably be detected in saliva and/or blood from the patients. We will do the detection of Periostin in the serum of patients with OSCC in the future. In conclusion, our studies have demonstrated a critical role of Periostin in invasion and angiogenesis of the metastatic process. These findings provide new and important information on the progression of OSCC. Our present findings raise the possibility that it could be used as a molecular target in antimetastatic therapy of OSCC patients.

## Figures and Tables

**Table 1:**
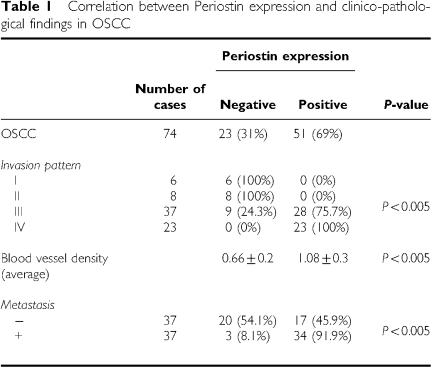
Correlation between Periostin expression and clinico-pathological findings in OSCC

## References

[bib1] Bao S, Ouyang G, Bai X, Huang Z, Ma C, Liu M, Shao R, Anderson RM, Rich JN, Wang XF (2004) Periostin potently promotes metastatic growth of colon cancer by augmenting cell survival via the Akt/PKB pathway. Cancer Cell 5: 329–339 15093540 10.1016/s1535-6108(04)00081-9

[bib2] Bishop ET, Bell GT, Bloor S, Broom IJ, Hendry NFK, Wheatley DN (1999) An *in vitro* model of angiogenesis: basic features. Angiogenesis 3: 335–344 14517413 10.1023/a:1026546219962

[bib3] Elkins T, Hortsch M, Bieber AJ, Snow PM, Goodman CS (1990) *Drosophila* fasciclin I is a novel homophilic adhesion molecule that along with fasciclin III can mediate cell sorting. J Cell Biol 110: 1825–1832 2335571 10.1083/jcb.110.5.1825 PMC2200178

[bib4] Fidler IJ (1991) Critical factors in the biology of human cancer metastasis: Twenty-eighth GHA Clowes Memorial Award Lecture. Cancer Res 50: 6130–6138 1698118

[bib5] Forastiere A, Koch W, Trotti A, Sidransky D (2001) Head and neck cancer. N Engl J Med 345: 1890–1900 11756581 10.1056/NEJMra001375

[bib6] Fox SB, Leek RD, Weekes MP, Whitehouse RM, Gatter KC, Harris AL (1995) Quantitation and prognostic value of breast cancer angiogenesis: comparison of microvessel density, Chalkley count and computer image analysis. J Pathol 177: 275–283 8551390 10.1002/path.1711770310

[bib7] Friedlander PL (2003) The use of genetic markers in the clinical care of patients with head and neck cancer. Arch Otolaryngol Head Neck Surg 129: 363–366 12622551 10.1001/archotol.129.3.363

[bib8] Gillan L, Matei D, Fishman DA, Gerbin CS, Karlan BY, Chang DD (2002) Periostin secreted by epithelial ovarian carcinoma is a ligand for v3 and v5 integrins and promotes cell motility. Cancer Res 62: 5358–5364 12235007

[bib9] Gonzalez HE, Gujrati M, Frederick M, Henderson Y, Arumugam J, Spring PW, Mitsudo K, Kim HW, Clayman GL (2003) Identification of 9 genes differentially expressed in head and neck squamous cell carcinoma. Arch Otolaryngol Head Neck Surg 129: 754–759 12874078 10.1001/archotol.129.7.754

[bib10] Horiuchi K, Amizuka N, Takeshita S, Takamatsu H, Katsuura M, Ozawa H, Toyama Y, Bonewald LF, Kudo A (1999) Identification and characterization of a novel protein, Periostin, with restricted expression to periosteum and periodontal ligament and increased expression by transforming growth factor beta. J Bone Miner Res 14: 1239–1249 10404027 10.1359/jbmr.1999.14.7.1239

[bib11] Jacobsson PA, Eneroth GM, Killander D, Moberger G, Martensson B (1973) Histologic classification and grading of malignancy in carcinoma of the larynx. Acta Radiol 12: 1–7 10.3109/02841867309131085 4725642

[bib12] Kalebic T, Williams JE, Talmadge JE, Kao-Shan CS, Kravitz B, Locklear K, Siegal GP, Liotta LA, Sobel ME, Steeg PS (1998) A novel method for selection of invasive tumor cells: derivation and characaterization of highly metastatic K1735 melanoma cells based on *in vitro* and *in vivo* invasive capacity. Clin Exp Metast 6: 301–318 10.1007/BF01753577 3359713

[bib13] Kim JE, Jeong HW, Nam JO, Lee BH, Choi JY, Park RW, Park JY, Kim IS (2002) Identification of motifs in the fasciclin domains of the transforming growth factor-*β*-induced matrix protein *β*ig-h3 that interact with the *α*v*β*5 integrin. J Biol Chem 277: 46159–46165 12270930 10.1074/jbc.M207055200

[bib14] Kim JE, Kim SJ, Lee BH, Park RW, Kim KS, Kim IS (2000) Identification of motifs for cell adhesion within the repeated domains of transforming growth factor-*β*-induced gene, *β*ig-h3. J Biol Chem 275: 30907–30915 10906123 10.1074/jbc.M002752200

[bib15] Kudo Y, Kitajima S, Sato S, Ogawa I, Miyauchi M, Takata T (2003) Establishment of an oral squamous cell carcinoma cell line with high invasive and p27 degradation activity from lymph node metastasis. Oral Oncol 39: 515–520 12747977 10.1016/s1368-8375(03)00015-0

[bib16] Kudo Y, Kitajima S, Ogawa I, Hiraoka M, Salgolzaei S, Keikhaee MR, Sato S, Miyauchi M, Takata T (2004) Invasion and metastasis of oral cancer cells require methylation of E-cadherin and/or degradation of membranous *β*-catenin. Clin Cancer Res 10: 5455–5463 15328184 10.1158/1078-0432.CCR-04-0372

[bib17] Kudo Y, Ogawa I, Kitajima S, Kitagawa M, Kawai H, Gaffney PM, Miyauchi M, Takata T (2006) Periostin promotes invasion and anchorage-independent growth in the metastatic process of head and neck cancer. Cancer Res 66: 6928–6935 16849536 10.1158/0008-5472.CAN-05-4540

[bib18] Nam JO, Kim JE, Jeong HW, Lee SJ, Lee BH, Choi JY, Park RW, Park JY, Kim IS (2003) Identification of the *α*v*β*3 integrin-interacting motif of *β*ig-h3 and its anti-angiogenic effect. J Biol Chem 278: 25902–25909 12704192 10.1074/jbc.M300358200

[bib19] Parkin DM, Bray F, Ferlay J, Disani P (2005) Global cancer statistics 2002. CA Cancer J Clin 55: 74–108 15761078 10.3322/canjclin.55.2.74

[bib20] Regezi JA, Sciubba JJ (1989) Ulcerative conditions: clinical–pathologic correlations. In Oral Pathology Regezi JA, Sciubba JJ (eds), pp 70–83. Philadelphia: WB Saunders

[bib21] Sasaki H, Auclair D, Kaji M, Fukai I, Kiriyama M, Yamakawa Y, Fujii Y, Chen LB (2001a) Serum level of the periostin, a homologue of an insect cell adhesion molecule, in thymoma patients. Cancer Lett 172: 37–42 11595127 10.1016/s0304-3835(01)00633-4

[bib22] Sasaki H, Dai M, Auclair D, Fukai I, Kiriyama M, Yamakawa Y, Fujii Y, Chen LB (2001b) Serum level of the periostin, a homologue of an insect cell adhesion molecule, as a prognostic marker in nonsmall cell lung carcinomas. Cancer 92: 843–848 11550156 10.1002/1097-0142(20010815)92:4<843::aid-cncr1391>3.0.co;2-p

[bib23] Sasaki H, Lo KM, Chen LB, Auclair D, Nakashima Y, Moriyama S, Fukai J, Tam C, Loda M, Fujii Y (2001c) Expression of Periostin, homologous with an insect cell adhesion molecule, as a prognostic marker in non-small cell lung cancers. Jpn J Cancer Res 92: 869–873 11509119 10.1111/j.1349-7006.2001.tb01174.x PMC5926835

[bib24] Sasaki H, Yu CY, Dai M, Tam C, Loda M, Auclair D, Chen LB, Elias A (2003) Elevated serum periostin levels in patients with bone metastases from breast but not lung cancer. Breast Cancer Res Treat 77: 245–252 12602924 10.1023/a:1021899904332

[bib25] Sawair FA, Irwin CR, Gordon DJ, Leonard AG, Stephenson M, Napier SS (2003) Invasive front grading: reliability and usefulness in the management of oral squamous cell carcinoma. J Oral Pathol Med 32: 1–9 12558952 10.1034/j.1600-0714.2003.00060.x

[bib26] Shao R, Bao S, Bai X, Blanchette C, Anderson RM, Dang T, Gishizky ML, Marks JR, Wang XF (2004) Acquired expression of periostin by human breast cancers promotes tumor angiogenesis through up-regulation of vascular endothelial growth factor receptor 2 expression. Mol Cell Biol 24: 3992–4003 15082792 10.1128/MCB.24.9.3992-4003.2004 PMC387763

[bib27] Tai IT, Dai M, Chen LB (2005) Periostin induction in tumor cell line explants and inhibition of *in vitro* cell growth by anti-periostin antibodies. Carcinogenesis 26: 908–915 15731169 10.1093/carcin/bgi034

[bib28] Takeshita S, Kikuno R, Tezuka K, Amann E (1993) Osteoblast-specific factor 2: cloning of a putative bone adhesion protein with homology with the insect protein fasciclin I. Biochem J 294: 271–278 8363580 10.1042/bj2940271 PMC1134594

[bib29] Unal OF, Ayhan A, Hosal AS (1999) Prognostic value of p53 expression and histopathological parameters in squamous cell carcinoma of oral tongue. J Laryngol Otol 113: 446–450 10505159 10.1017/s0022215100144184

